# Solute Carrier Family 26 Member a2 (*slc26a2*) Regulates Otic Development and Hair Cell Survival in Zebrafish

**DOI:** 10.1371/journal.pone.0136832

**Published:** 2015-09-16

**Authors:** Fei Liu, Wenjun Xia, Jiongjiong Hu, Yingzhi Wang, Fan Yang, Shaoyang Sun, Jin Zhang, Nan Jiang, Huijun Wang, Weidong Tian, Xu Wang, Duan Ma

**Affiliations:** 1 Key Laboratory of Metabolism and Molecular Medicine, Ministry of Education, Collaborative Innovation Center of Genetics and Development, Department of Biochemistry and Molecular Biology, Institute of Medical Sciences, School of Basic Medical Sciences, Fudan University, Shanghai, 200032, China; 2 Institutes of Biomedical Science, Fudan University, Shanghai, 200032, China; 3 Department of Otorhinolaryngology, Shanghai East Hospital, Tongji University, Shanghai, China; 4 Children’s Hospital, Fudan University, Shanghai, 200032, China; 5 School of Life Sciences, Institute of Biostatistics, Fudan University, Shanghai, P.R. China; Universitat Pompeu Fabra, SPAIN

## Abstract

Hearing loss is one of the most prevalent human birth defects. Genetic factors contribute to the pathogenesis of deafness. It is estimated that one-third of deafness genes have already been identified. The current work is an attempt to find novel genes relevant to hearing loss using guilt-by-profiling and guilt-by-association bioinformatics analyses of approximately 80 known non-syndromic hereditary hearing loss (NSHL) genes. Among the 300 newly identified candidate deafness genes, slc26a2 were selected for functional studies in zebrafish. The slc26a2 gene was knocked down using an antisense morpholino (MO), and significant defects were observed in otolith patterns, semicircular canal morphology, and lateral neuromast distributions in morphants. Loss-of-function defects are caused primarily by apoptosis, and morphants are insensitive to sound stimulation and imbalanced swimming behaviours. Morphant defects were found to be partially rescued by co-injection of human SLC26A2 mRNA. All the results suggest that bioinformatics is capable of predicting new deafness genes and this showed slc26a2 is to be a critical otic gene whose dysfunction may induce hearing impairment.

## Introduction

Hearing impairment is one of the most common losses of meaningful function in humans, and it poses a persistent threat to worldwide public health. Approximately 10% of people worldwide have mild or moderate hearing impairment [1]. Genetic factors are important to the pathogenesis of deafness, and ≈80% of genetic deafness is non-syndromic hearing impairment (NSHI). Historically, most deafness-causing genes have been identified in inherited deafness pedigrees by linkage analysis, but similar pedigrees have been difficult to find in modern society. However, broad application of 2nd-generation sequencing has facilitated the search for deafness genes at a genome-wide scale [2]. Currently, 170 mutation loci and nearly 80 genes have been identified in NSHI patients, but another two-thirds of deafness genes have yet to be discovered [3]. Identification of these unknown genes is necessary if clinicians are to diagnose and treat syndromic and non-syndromic deafness.

Current methods of screening newly identified disease-causing genes are expensive and time-consuming. Recent developments in bioinformatics offer unique and convenient approaches. Various network-based approaches have been employed for exploring genotype-to-phenotype relationships, and the research found that protein products of genes associated with similar diseases are more likely to physically interact and form disease-specific functional modules [4–5]. On the basis of this easily accepted “guilt-by-association” principle, many methods have been developed for the prediction of novel disease-associated genes with the use of the protein interactome network [6–7]. However, most accurate functional annotation via homology analysis depends on sequence conservation of target proteins, and many homologous proteins have unknown functions. For this reason, a second method is used to determine gene function based on characteristic profiles (guilt-by-profiling) [8]. The sequence-based guilt-by-association method predicts gene function based on multiple alignments of conserved motifs and domains with known proteins, and the guilt-by-profiling method predicts gene functions via the mining of published databases [9]. Combining these methods allows us to significantly improve component classifiers specificity and synergy.

To discover new deafness genes, guilt-by-profiling and guilt-by-association analyses of 77 reported NSHL genes were combined. Ultimately, *SLC26A2* was chosen for study because it met the selection criteria. SLC26A2 is a diastrophic dysplasia sulphate transporter necessary for proteoglycan synthesis in the bone and cartilage extracellular matrix and for expansion of chondrocyte volume [10–11]. SLC26A2 has been reported to be a SO4^2-^/Cl^-^/OH^-^ exchanger and it is precisely regulated by extracellular Cl^-^ [12]. Mutations in four human SLC26 genes have been found to be associated with congenital and early-onset Mendelian diseases: chondrodysplasias (*SLC26A2*), chloride diarrhea (*SLC26A3*), and deafness with enlargement of the vestibular aqueduct (*SLC26A4*) [13]. *SLC26A2* is highly conserved among different vertebrate species and zebrafish *slc26a2* protein shares 87.6% identical amino acid sequence with human *SLC26A2* protein, which consists chiefly of a sulphate transporter domain, the STAS domain, and the C terminal dimerization domain, which are key functional domains shared with the remaining Slc26 family members.

Zebrafish are excellent for the study of the development of deafness because they have two sensory organs that detect changes in the water: the inner ear and the lateral line system. Zebrafish have no outer or middle ear but rather a typical vertebrate inner ear composed of otoliths, semicircular canals. Zebrafish have five sensory organs as larvae but seven as adult. The larval ones are three cristae and two maculae (saccular and utricular) while the adult adds the lagenar macula and macula neglecta [14], and the sensory cristae containing supporting cells and hair cells [15–16].

Degeneration and death of inner ear sensory hair cells is an underlying cause of hearing loss [17], and this is usually permanent, mammalian inner ear hair cells cannot regenerate [18]. In addition, zebrafish have a series of mechanosensory neuromasts on the body lateral line surfaces, and those units are structurally and functionally similar to sensory patches of the inner ear [16]. The lateral line system is initiated and developed through the mutual interaction of the FGF and Wnt signalling pathways [19–20]. Each neuromast consists of chrysanthemum-like hair cells in a framework of supporting cells, and hair cell kinocilia are encased in a gelatinuos copula, which can be deflected by water waves [20]. In this way, this model facilitated study of *slc26a2* in hearing development.

To understand the role of *slc26a2* in the pathogenesis of deafness, its expression was knocked down in zebrafish through morpholino injection and changes in zebrafish behaviour were observed. Results suggested severely disturbed hearing and balance in slc26a2-knockdown animals. Injection of slc26a2 antisense morpholino oligonucleotides(MO) resulted in abnormal types of otoliths, altered numbers of otoliths, malformed semicircular canals in the inner ear, and reduced numbers of lateral line neuromasts. Then a rescue experiment was conducted. Results showed that the knockdown phenotype could be partially corrected after co-injection with human *SLC26A2*-mRNA. These findings suggest that *slc26a2* is essential to inner ear and lateral line neuromast development and to maintenance of normal hearing and balance in zebrafish. This work also confirms that bioinformatics can be used to predict deafness genes and prioritize experimental studies of the functions of deafness genes.

## Materials and Methods

### 2.1. Screening of candidate deafness gene by bioinformatics

**Fig 1:**
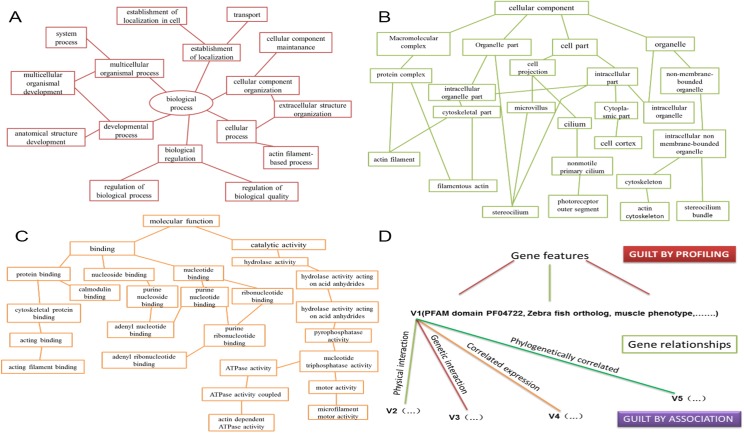
Guilt-by-profiling and guilt-by-association point selection method for a given function probability value calculation. Guilt-by-association sorting is based on gene function contact (FL). Each FL division has a weight value and some probability that two genes annotated with the same GO pathway. GO annotations are classified within a given range. All 12 categories of GO pathways produced specific FL maps and each FL map corresponded to at least one diagram with these GO branches: (A) biological processes, (B) cellular components, and (C) molecular functions. We scored combinations of specific genes and GO annotations using the FL map. (D) Guilt-by-profiling and guilt-by-association algorithm scores were obtained using a logistic regression model combined with free parameters.

Guilt-by-profiling exploits correlation between function and other gene characteristics and guilt-by-association transfers function from one gene to another via biological relationships. Guilt-by-profiling and guilt-by-association were combined for analysis of features and functional interactions of purported deafness-causing genes [21]. These data were crovalidated with the literature. Sequence-based guilt-by-association was used to predict gene function using multiple conserved motif and domain alignments common to proteins known to be associated with hearing impairment. The guilt-by-profiling method was used to predict gene function using database mining. Using both methods significantly improved specificity and synergy of the component classifiers (Fig 1).

### 2.2. Zebrafish husbandry

AB line zebrafish were used and maintained in the Fudan University Zebrafish Breeding Centre. Tg (Brn3c:mGFP) S356T transgenic zebrafish expressing GFP in hair cells under control of the POU4F3 promoter that is targeted to the plasma membrane with a GFP-43 membrane targeting sequence (provided by Prof. Li Huawei, Fudan University). Embryos were raised in petri dishes at 28.5°C. Developmental stages were evaluated as hours and days post-fertilization (hpf and dpf). All zebrafish experimental protocols were approved by the Institutional Animal Care and Use Committee of Fudan University.

### 2.3. RT-PCR, whole-mount RNA in situ hybridization in zebrafish

Reverse transcription PCR was used to detect expression of *slc26a2* at different time points in zebrafish development. Slc26a2: 5′-CGTCAGACATCAATCAGACCACC-3′ (forward) and 5′-TGACCGTGAAACAATGAAAGTAG-3′ (reverse). The housekeeping gene GAPDH, forward primer 5′-CCCAATGTCTCTGTTGTGGA-3′ and reverse primer 5′-GGTCACATACACGGTTGCTG-3′ were used as an internal control to produce a 275 bp product. Whole mount in situ hybridization (WISH) was used with digoxigenin-labelled antisense cRNA probes according to the protocol of the manufacturer (Roche Applied Science, Mannheim, Germany) with *slc26a2* DNA templates. WISH of albino embryos was performed as described [21]. Hybridized probes were detected with alkaline phosphate-conjugated anti-DIG antibody and visualized with NBT/BCIP.

### 2.4. Gene knockdown and rescue in zebrafish

There are two transcripts of *slc26a2* in zebrafish, we designed morpholinos of *slc26a2a* and *slc26a2b* and injected them into zebrafish one cell fertilized eggs, respectively. Results showed that knockdown of *slc26a2a* had no effects on development of semicircular canals and maturation of lateral line neuromasts. Thus, though there are two *slc26a2* paralogs in zebrafish, effects of *slc26a2* on hearing can be entirely indicated by *slc26a2b* only. Therefore, in the following experiments, only *slc26a2b* was knocked down to study the function of *slc26a2* in zebrafish hearing.

The *slc26a2* gene was knocked down using a single specific antisense morpholino oligonucleotides (MOs). The antisense oligonucleotide modified pre-mRNA splicing in the nucleus by targeting splice junctions between intron1 and exon2 of *slc26a2* (S2-SP1) which led the loss of exon2 in mRNA. Mismatch controls (Mcon) allowed correct interpretation of morpholino experiments [22].

S2-SP1: 5ʹ-TTGGTTGCAGGTGTTGATGGGTCTG-3ʹ;

S2-SP1-Mcon: 5′-CCTTGAACAGCTCCAACGAAATCAA-3′;

All MOs were obtained from Gene Tools (Philomath, OR, U.S.).

RT-PCR was performed on the cDNA samples isolated from 3dpf morphants and WT zebrafish, and the sequences of the primers used are: 5′-CAGGCCTTATTGTTGGCATT-3′ (forward, in exon1) and 5′-AGGCAGAAACCACAACTCCA-3′ (reverse, in exon 3). The RT-PCR performed on wildtype cDNA produces a 1256bp band for slc26a2, while the RT-PCR performed on S2-SP1 morphant cDNA yields another 516bp band in addition to the 1256bp band. The housekeeping gene gapdh was used as an internal control to produce a 275 bp product.

Both slc26a2 MOs and Mcon of slc26a2 were diluted to the indicated concentrations with RNase-free water, and then injected into one-cell stage zebrafish embryos. S2-SP1 could effectively downregulate slc26a2 expression. We chose a representative morphine S2-SP1 for display. For rescue studies, MOs were co-injected into 1-cell-stage zygotic with human SLC26A2 mRNA. Human *SLC26A2* mRNA was synthesized *in vitro* from linearized plasmids using the T7 Ultra Kit (Ambion, Austin, TX, U.S.) according to the manufacturer’s instructions.

### 2.5. AO staining

MOs is effective for inducing sequence-specific gene knockdown in multiple systems; however, MO can induce off-target effects chiefly mediated through p53 activation [23]. Concurrent knockdown of p53 specifically ameliorates cell death induced by MO off-targeting [24]. To exclude the possibility that the otic disorder and decreased hair cells of MO knockdown zebrafish were due to off-target MO toxicity, we co-injected S2-SP1 (4 ng) with p53-MO (4 ng). Apoptosis in whole zebrafish larvae was measured with vital dye acridine orange staining [25]. Live embryos were immersed in 5 μg/ml acridine orange (Sigma) dissolved in PBS in the dark for 30 min. Staining was then visualized and imaged for approximately 60 s (signal quenched after 60 s exposure to fluorescence) with a fluorescent microscope (DP70, Olympus, Tokyo, Japan).

### 2.6. FM1-43FX, 4′,6-diamidino-2-phenylindole (DAPI), and phalloidin staining

Neuromast hair cells were labelled by exposing live 120hpf larvae to 2.5 μM FM1-43FX for 1 min 30 s in the dark. Larvae were quickly rinsed five times in PBS, and labelled larvae were anesthetized with 3-aminobenzoic acid ethyl ester methane sulphonate (MS-222) and fixed in 4% paraformaldehyde (PFA) overnight at 4°C. Then, DAPI nuclear staining was used to quantify hair cells. Labelled hair cells of neuromasts along the body were recorded on one side of each fish and visualized by confocal microscopy with a 63× oil lens. Cilia in 120hpf zebrafish inner ear were marked with 2.5 mg/ml fluorescein isothiocyanate (FITC)-labelled phalloidin (Sigma) in PBS at room temperature. Light exposure was avoided after fixation in 4% PFA, 2% Triton X-100 (Sigma) in PBS. Finally, embryos were washed several times in PBS for over 2 h and viewed under a confocal microscope.

### 2.7. Terminal dexynucleotidyl transferase (TdT)-mediated dUTP nick end labelling (TUNEL) assay

First, 120hpf embryos were fixed in 4% PFA for 1 h at room temperature, and then washed twice with PBS buffer and permeabilized with 0.1% sodium citrate and 0.1% TritonX for 2 min. After being washed twice in PBS buffer, embryos were incubated with a reaction mixture containing TMR-labelled nucleotides and terminal deoxynucleotidyl transferase for 30 min in the dark at 37°C. The reaction was stopped by washing five times with PBS. Terminal deoxynucleotidyl transferase catalysed the incorporation of labelled nucleotides to 3′OH DNA ends in a template-independent reactions. Fluorescence was visualized and imaged under a confocal microscope (TCS SP8, Leica).

### 2.8. Immunofluorescence

Tg (Brn3c:mGFP) S356T transgenic zebrafish were fixed at 120hpf in 4% PFA overnight, and then washed twice by PBS buffer. After the PBS wash, the embryos were digested by collagenaseⅡfor 2 hours, and washed twice again in PBS. Embryos were permeabilized in PBS with 0.5% Triton X-100 (Sigma) 5 times for 15 min, and refixed in 4% PFA. 10% goat serum (Sigma) was used to block nonspecific binding, and the rabbit anti-sox2(a member of the SRY-related HMG-box (SOX) family of transcription factors) antibody and goat-anti-rabbit Cy3 antibody were both used at 1:1000.

### 2.9. M-cell recording and startle response tests

The inner ear is crucial to zebrafish hearing and balance [26]. Because predominant defects in the inner ears of MO-knockdown zebrafish have been reported, swimming behaviour and hearing were investigated in MO zebrafish. To test the hearing ability, we tested the M-cell recording and fast escape reflex, the C-shaped startle response mediated predominately by M-cells, using near-field pure tone stimulation with two different levels of sound intensity [27–28]. Embryos were raised in Hank’s solution with 100 mM phenylthiourea (Sigma), and 120hpf larvae were paralyzed with 0.1% a-bungarotoxin (Tocris Bioscience) and mounted in 1% low melting agarose gel for M-cell recording. Breakthrough whole-cell recording of M-cells was made under visual control. The micropipette was made from borosilicate glass capillaries. The internal solution contained 110 mM K-gluconate, 6 mM NaCl, 2 mM MgCl2, 2 mM CaCl2, 10 mM HEPES, 10 mM ethylene glycoltetraacetic acid (pH 7.3) and the reversal potential of chlorideion (ECl2) was about 260 mV. Recording was made withpatch-clamp amplifiers (MultiClamp 700B, Axon Instruments). For sound stimulation, brief pure tones (10 ms,500 Hz) with several intensities were given through the air from a voice box (Edifier, R1800TII)[29]. The C-shaped startle response was tested in 96-well plastic plates and recorded with a high-speed camera (Redlake, MotionScope M3, 1000 fps) under infrared light illumination. Pure tone stimulations (10 ms, 500 Hz) of two different intensities were given through a plastic board mounted on a voice box (HiVi, D1080MKII). Each larva was tested 14–16 times and the relative number of C-startle times was calculated for each larva.

### 2.10. Statistical Analysis

Data are shown as means ± SEM. All data were processed by SPSS, mono factor analysis of variance was used to analysis. Difference was considered statically significant at *P*<0.01.

## Results

### 3.1. Candidate deafness gene screening

Here, 77 reported deafness genes were classified according to their biological characteristics, and the categorization system was combined with 2,455 gene ontology (GO) terms. More than 300 deafness candidate genes were predicted according to the features of 16 gene types and 6 class interaction relationships among different genes based on analysis and simulations using the Funckenstein algorithm. Candidate genes were ranked according to rating system quality and deafness candidate genes were predicted. Relevant literature was found in PUBMED, and publications were using the following selection criteria: Genes must 1) rank high among candidate genes; 2) have gene family members that are known deafness genes; 3) be expressed in the cochlea, epithelium, or cilia of mammals; 4) have been studied in whole-animals model (not only in cells); and 5) be related to the development of the auditory system. Here, 17 candidate genes were found to meet these criteria (Table 1). Of these, top-ranked *slc26a2* was chosen because of its most homology between humans and zebrafish.

**Table 1:** Top ranked candidate deafness genes having physical interaction with hearing loss genes.

Gene	Location	Gene family members that are known deafness genes	Expression organs	Cell/Animal research
SLC26A2	5q431-q34	SLC26A5	Multiple tissues	Mouse
		SLC26A4		
		MYO7A	Inner ear	
MYO7B	2q21.1	MYO3A	Kidney	NA
MYO10	5p15.1-p14.3	MYO6	Testis	Mouse
			Epidermis	
TMC3	15q25.1	TMC1	Inner ear	NA
LHFPL3	7q22.2	LHFPL5	Bone	NA
GRXCR1	4p13	GRXCR1	Inner ear	Mouse
			Retinal	
			ganglion	
POU4F2	4q31.2	POU4F3	cells	Mice
LHFPL4	3p25.3	LHFPL5	NA	NA
DIAPH2	Xq21.33	DIAPH1	NA	NA
		USH1G		
		USH1F		Epithelial
USH1G	17q25.1	USH1C	Retina	cells
			Epidermis	
			Lung kidney	
GRHL3	1p36.11	GRHL2	Heart	Null mice
KCNQ2	20q13.3	KCNQ4	Brain	Zebrafish
POU4F1	13q31.1	POU4F3	Somatosensory neurons	Mice
		GJB2	Heart	
		GJB3	Lactating mammary gland	
GJB1	Xq13.1	GJB6	Postnatal brain	Mice
				Mice
GRHL1	2p25.1	GRHL2	Epidermis	Zebrafish
POU3F3	2q12.1	POU4F3	NA	NA
		MYH9	Leukemia	Knocked-in
MYH11	16p13.11	MYH14	Bone marrow	mouse
TMC8	17q25.3	TMC1	Epidermis	NA
MYO15B	17q25.1	MYO15A	NA	NA

### 3.2. Slc26a2 is expressed throughout embryonic development

**Fig 2:**
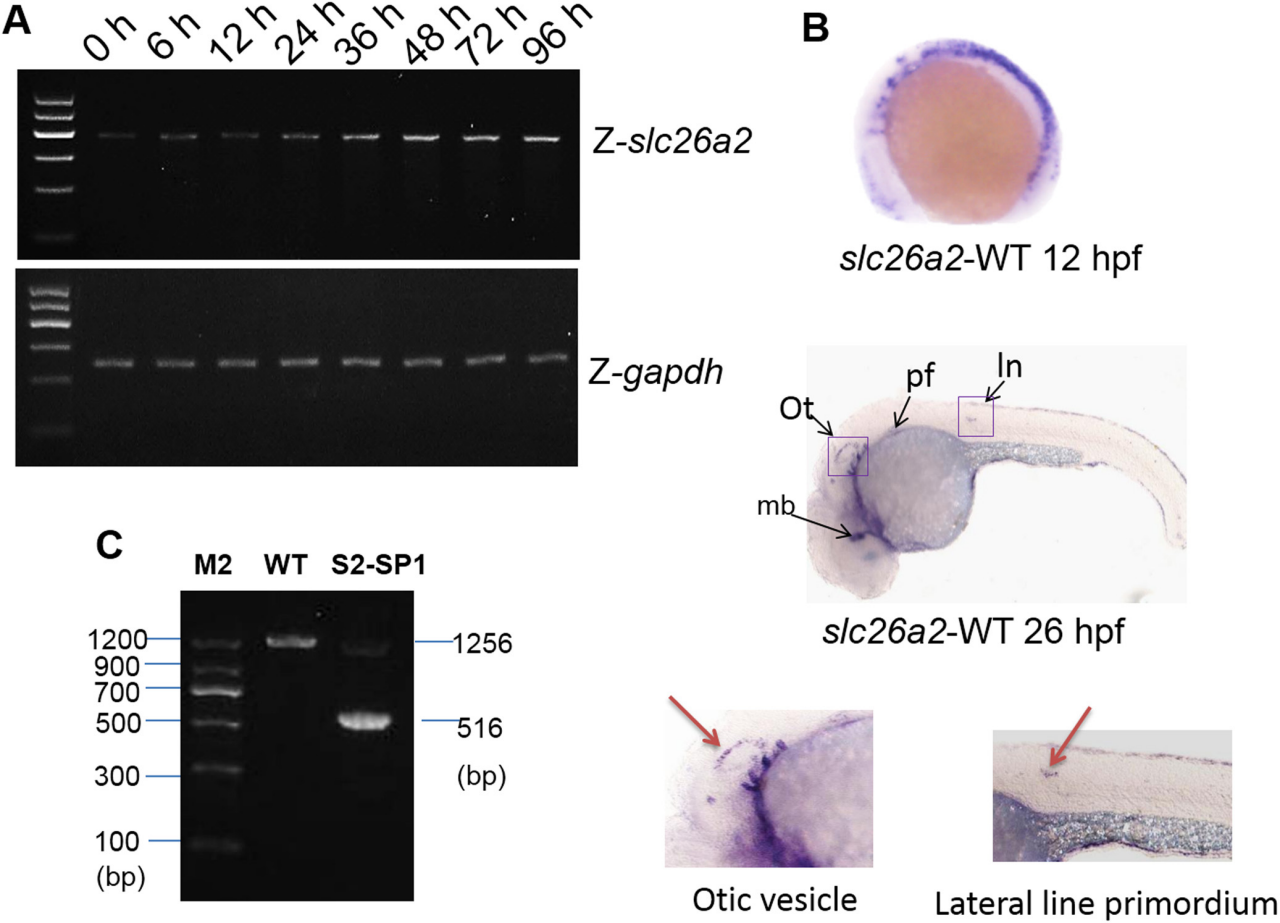
*slc26a2* mRNA expression in embryonic and adult zebrafish. (A)RT-PCR analysis of *slc26a2* expression in 0–96 h zebrafish yielded 405 bp products, GAPDH served as an internal control for cDNA quantification and gave a 275 bp product. mRNA expression of *slc26a2* was detected in the first development period, and transcription remained high after 48 h. (B) Expression of *slc26a2* in WT embryos at 12 and 26hpf, detected by in situ hybridization, at 12hpf. The *slc26a2* transcript was expressed broadly, and at 26hpf, *slc26a2* was expressed circumferentially around the ln, lateral line primordium; ot, otic vesicle; pf, pectoral fin, and mb, midbrain. This suggests a function for slc26a2 in the inner ear and neuromast development. Wild insets depict enlarged images of the otic vesicles and lateral neuromasts. (C) RT-PCR confirmed the effectiveness of S2-SP1, and specific products were amplified from cDNA. Wild-type zebrafish yielded 1256 bp products for *slc26a2*, and morphants had 516 bp products for S2-SP1 in addition to those observed in WT fish. Two bands appeared for morphants but only one band was seen for WT.

To characterize *slc26a2* developmental expression, temporal expression patterns of *slc26a2* were examined during embryogenesis using RT-PCR. As shown in (Fig 2A), the maternal transcript was present from the one-cell stage and remained until zygotic expression was initiated. Gene expression gradually increased from 36 to 96 h post-fertilization (hpf).

To characterize developmental expression of *slc26a2*, *in situ* hybridization using *slc26a2* antisense probes was performed, and the *slc26a2* expression pattern in WT embryos is indicated in (Fig 2B). At 12hpf, *slc26a2* transcripts are expressed broadly, but at 26hpf, *slc26a2* expression was around the lateral line primordium, otic vesicle, pectoral fin, and midbrain, suggesting that *slc26a2* may contribute to hearing development.

### 3.3. Developmental processes involved in inner ear was delayed in Slc26a2-morphant zebrafish

*RT-PCR* was used to confirm the efficacy of MO splicing. Specific products were amplified from cDNA of the 120hpf morphant and WT zebrafish, and 1,256 bp products were obtained for WT zebrafish, but 516 bp products were obtained from morphant S2-SP1 in addition to products that were identical to WT samples (Fig 2C). These data suggest that S2-SP1 disrupts normal splicing of *slc26a2* primary transcripts and knock down *slc26a2* effectively.

**Fig 3:**
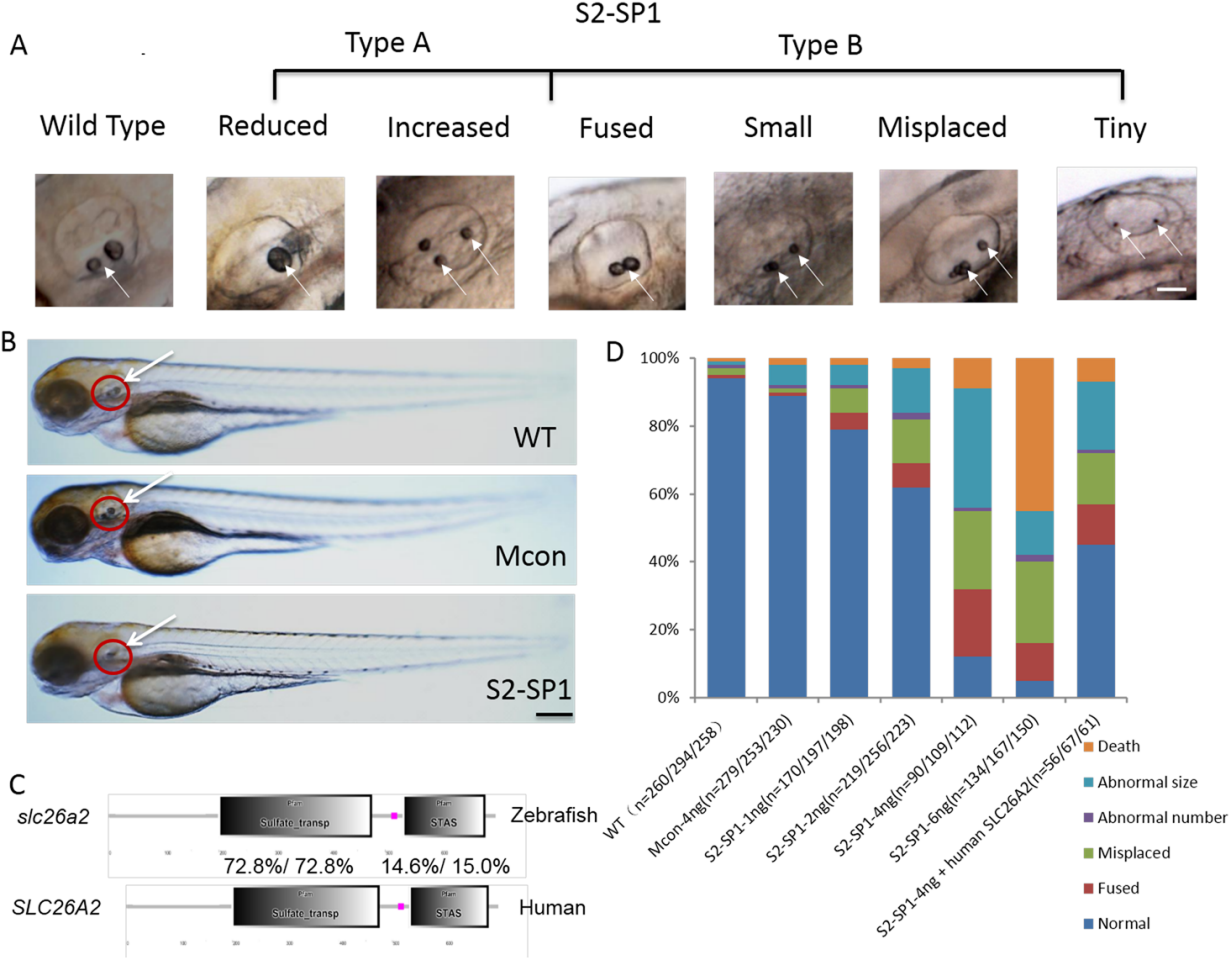
*slc26a2* knockdown in wild-type embryos and rescue of *slc26a2* overexpression in S2-SP1 morphants. (A) Variations in otolith size of morphant (S2-SP1 knockdown) embryos were observed at 72hpf. Based on the otolith size, malformed embryos were classified as type A (abnormal phenotype, including small, tiny, fused and misplaced otoliths) or type B (abnormal number of otoliths, including increased and decreased otoliths). (B) Morphology of 72hpf WT embryos injected with different morpholinos. One or two cell embryos were injected with 4 ng of S2-SP1 or Mcon. WT. Note the change in otolith size. (C) Schematic representation of two functional domains of zebrafish and human *slc26a2* proteins. Degree of identity/similarity indicated for the sulphate transporter, STAS, and the C terminal dimerization domains. (D) Relative numbers of embryos in each category. Single-celled embryos were injected with indicated morpholinos at the indicated dose and categorized at 72hpf. Single-celled S2-SP1 embryos were either uninjected or were injected with 150 pg of human *SLC26A2* mRNA and categorized at 72 hpf. (N = number of observed embryos).

Evidence suggests that the inner ear is necessary to zebrafish hearing and balance, so morphological defects of the inner ear were measured in *slc26a2*-morphant zebrafish. The morphology of WT, Mcon, and S2-SP1-injected zebrafish was observed at 72hpf (Fig 3A). S2-SP1 zebrafish embryos were not abnormal (no spinal curvatures or pericardialites). Inner ear of morphants had otolith abnormalities (white arrow), and by 72hpf, most otoliths were of normal appearance in WT and Mcon embryos. In contrast, S2-SP1 morphants had small, fused, misplaced, and deranged numbers of otoliths, and the inner ear was smaller with a malformed semicircular canal. Except for the inner ear and nuromast abnormity, the differences of entirety morphology zebrafish among WT and morphants were not obviously (Fig 3B).

Quantitative analysis of otolith defects caused by S2-SP1 at different concentrations at 72hpf were measured and 4 ng was chosen as the optimal MO concentration for *slc26a2* (Fig 3D), this concentration was less lethal and offered the greatest ratio of abnormal phenotypes. Experiments were performed in triplicate. Co-injection S2-SP1 (4 ng) with p53 MO had no effect on apoptotic signalling (S1 Fig), confirming that MO-induced developmental defects are target-independent rather than off-target toxic effects.

**Fig 4:**
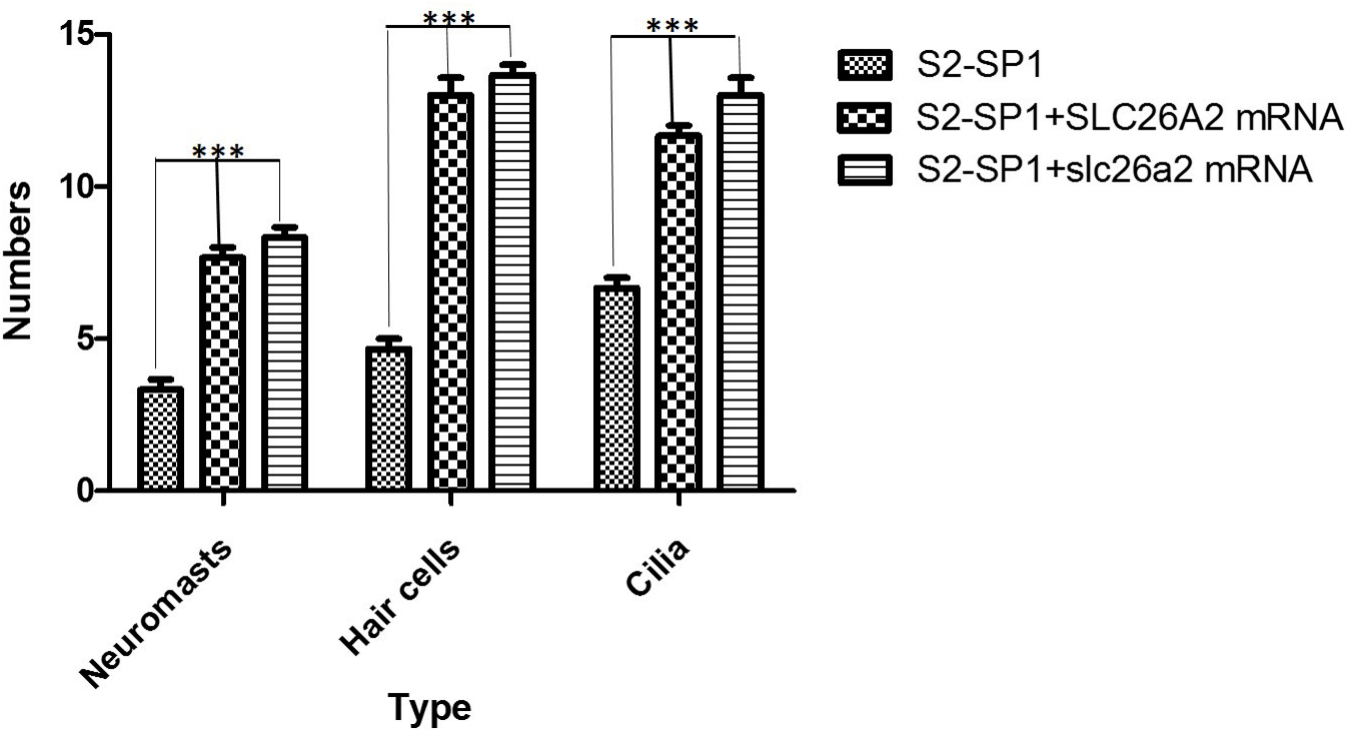
Zebrafish or human mRNA co-injection rescues the numbers of neuromasts, hair cells per neuromast and cilia per neuromast in morphants. The rescue experiments are performed by co-injecting S2-SP1 with SLC26A2 mRNA or slc26a2 mRNA, and the numbers of neuromasts, hair cells per neuromast and cilia per neuromast in the rescued embryos are all increased compared with S2-SP1 morphants alone. Neuromasts, hair cells and cilia were detected in 120hpf Tg (Brn3c:mGFP) transgenic zebrafish larvae. The average L1-L8 neuromasts of 120hpf zebrafish was 4.6 ± 0.4 for S2-SP1 zebrafish, while 7.6 ± 0.5 for S2-SP1+SLC26A2 mRNA, 7.8 ± 0.3 for S2-SP1+slc26a2 mRNA. Numbers of hair cells per neuromast both in anterior and posterior line in S2-SP1 knockdown 120hpf larvae were 4.5±0.5 less than in 120hpf zebrafish were 13.0 ±1.0 for S2-SP1+SLC26A2 mRNA and for S2-SP1+slc26a2 mRNA were 13.5 ± 0.5. And numbers of cilia in morphants were 6.6 ± 0.5 less than in 120hpf zebrafish were 11.5 ±0.5 for S2-SP1+SLC26A2 mRNA and for S2-SP1+slc26a2 mRNA were 13 ± 1. Statistical analysis of numbers of neuromasts, hair cells per neuromast and cilia in different types of embryos at 120hpf, including S2-SP1, S2-SP1+SLC26A2 mRNA and S2-SP1+slc26a2 mRNA. ***P<0.001.

Considering the high conservation of slc26a2\Slc26 family members in evolution (S2A and S2B Fig), human *SLC26A2* mRNA was co-injected with S2-SP1 into embryos at the one-cell stage to rescue abnormalities of zebrafish morphant. Results show that human *SLC26A2* mRNA could largely rescue MO phenotypes (Fig 3D). Injection of S2-SP1 caused an abnormal otic phenotype in 88% of zebrafish, and co-injection of human-*SLC26A2* mRNA with S2-SP1 resulted in a normal phenotype in 45% of these zebrafish. Zebrafish *slc26a2* mRNA was also co-injected with S2-SP1 into embryos at the one-cell stage. Results show that zebrafish *slc26a2* mRNA could rescue MO phenotypes as well (Fig 4).

### 3.4. Knockdown of *slc26a2* has distinct effects on cilia development and neuromast deposition

**Fig 5:**
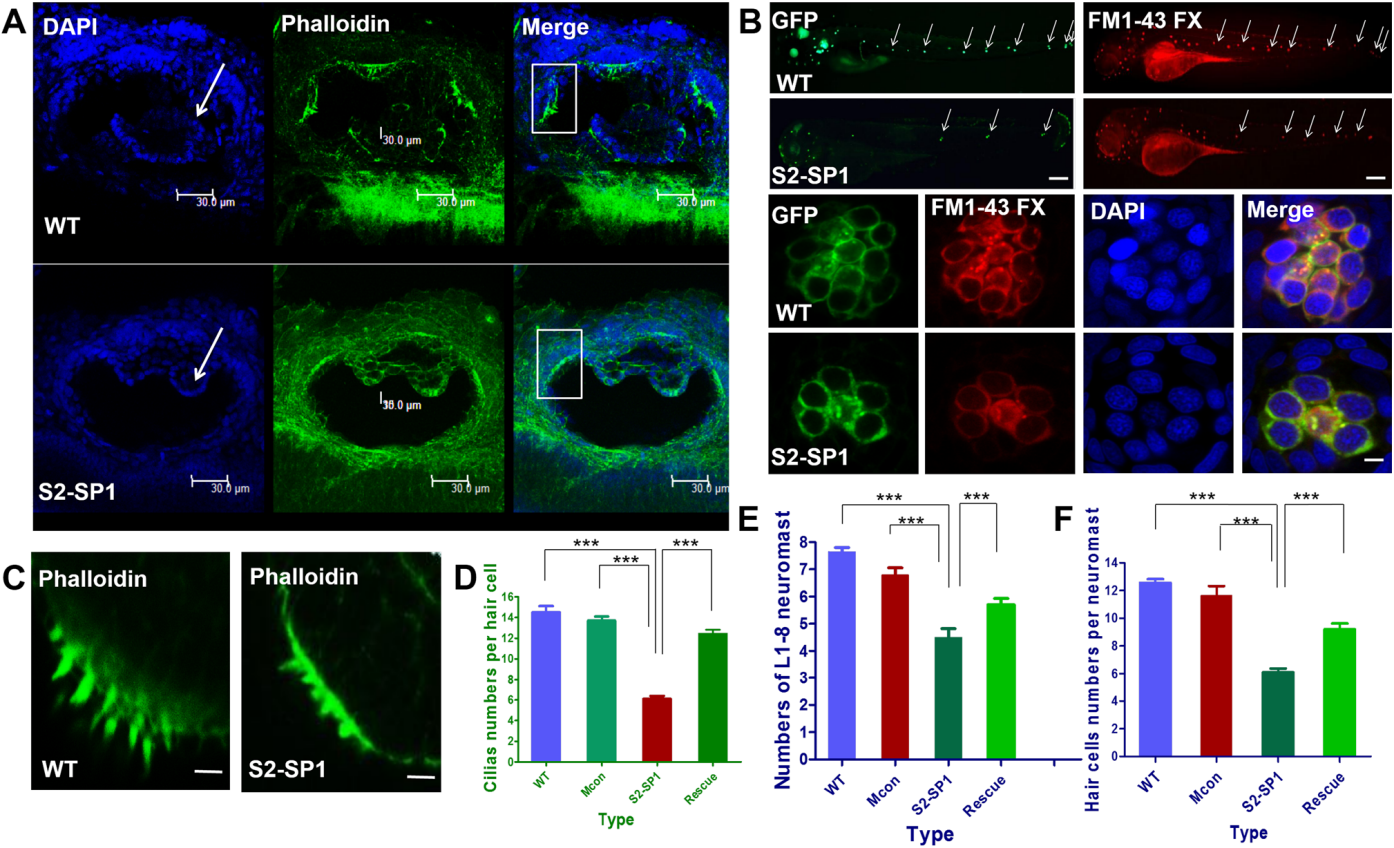
Knockdown of *slc26a2* induces semicircular canal defects in larval zebrafish. (A) Changes in semicircular canal morphology and cilia size (scale bars = 30 μm). (B) Changes of neuromasts numbers and hair cell numbers in lateral line (scale bars = 20 μm). (C, D) Changes of cilia numbers in the inner ear (scale bars = 20 μm). (D) Statistical analysis of the ciliary bundles in the inner ear in different types of embryos at 120hpf, including wild type(WT), mismatch-MO control, S2-SP1 MO and Rescue. (E) Statistical analysis of the L1-8 neuromasts numbers in the posterior lateral line in different types of embryos at 120hpf, including WT, Mis-con, S2-SP1 and Rescue. (F) Statistical analysis of the hair cells numbers per neuromast in different types of embryos at 120hpf, including WT, Miscon, S2-SP1 and Rescue. ***P<0.001.

The functional status of cell cilia was assessed, and cilia were stained 120hpf using phalloidin labelled with FITC. Data showed that S2-SP1 morphants had distinct semicircular canal defects that were not found in WT (Fig 5A). There were fewer variable stereocilia in S2-SP1 knockdown embryos at 120hpf than in WT zebrafish (scale bars = 5 μm). The average number of cilia in cristae was 6.7 ± 0.5 for S2-SP1, whereas in WT it was 14.3 ± 1.6 (Fig 5D).

The functional status of neuromast hair cells in lateral lines was observed by briefly exposing larvae to FM1-43, a styryl pyridinium dye, which enters the hair cells via partially open MET channels at rest [30]. Then, DAPI nuclear staining was used to quantify cells [31]. Injection with S2-SP1 led to decreased and disordered neuromasts in the lateral line (Fig 5B), neuromasts and hair cells were detected in 120hpf Tg (Brn3c:mGFP) transgenic zebrafish larvae. The average L1-L8 neuromasts of 120hpf zebrafish was 4.6 ± 0.5 for S2-SP1 zebrafish, while 7.7 ± 0.7 for WT (Fig 5F). There were also fewer hair cells per neuromast both in anterior and posterior line in S2-SP1 knockdown 120hpf larvae than in wild-type (Fig 5F), which had complete clusters of regularly arranged hair cells, average functional hair cells per neuromast in 120hpf zebrafish was 6.3 ± 1.0 for S2-SP1 and for WT was 12.5 ± 1.2. Then the development of somites in zebrafish was observed to assess if abnormal neuromast deposition was caused by abnormal development of somites. The somites of 72hpf and 120hpf zebrafish were counted and there were little differences between WT and morphants. As a result, there was no abnormal phenotype of somites when knocking down *slc26a2*.

### 3.5. Neuromast hair cell apoptosis in 120hpf larvae

**Fig 6:**
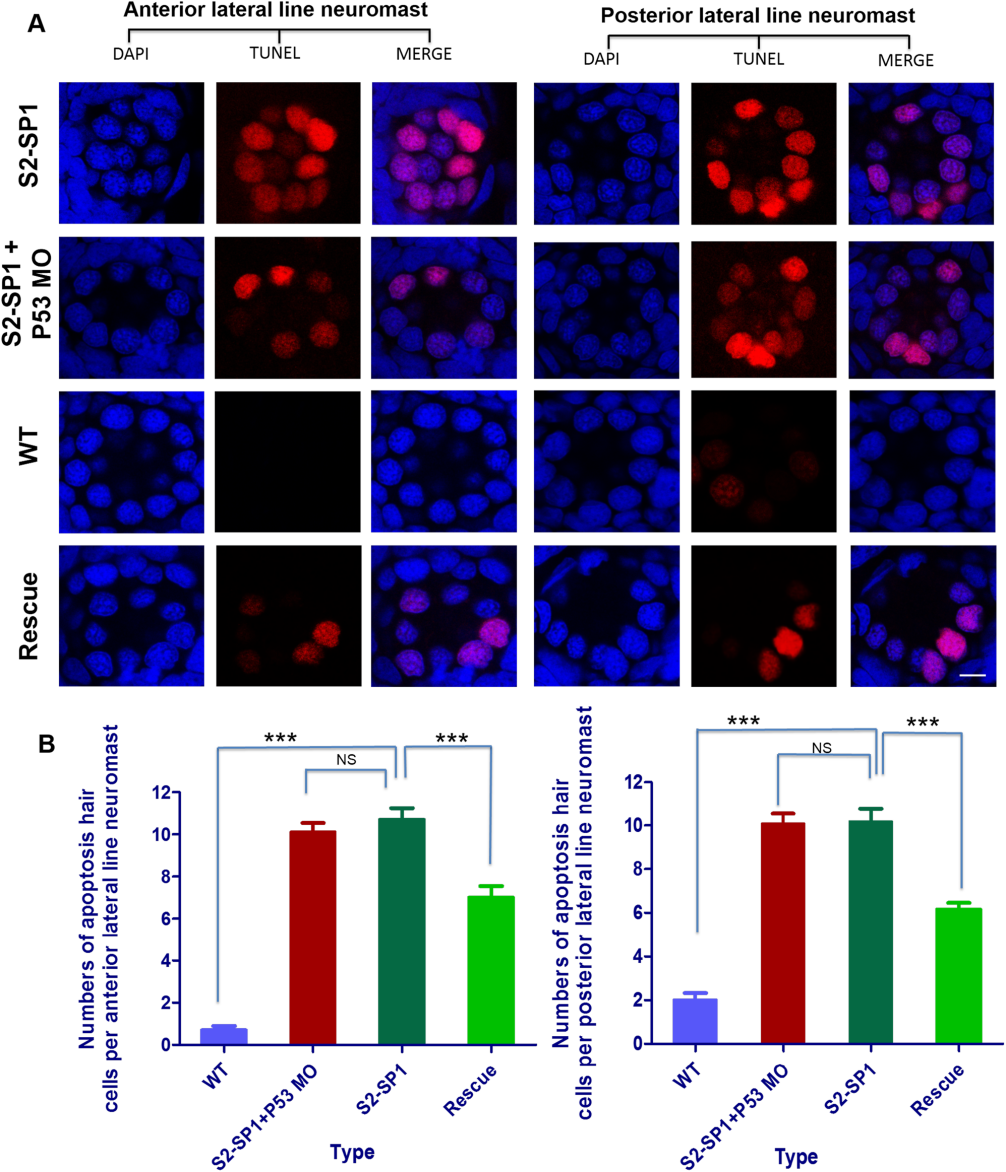
Knockdown of *slc26a2* induces apoptotic signals. Morphology of 120hpf WT AB line zebrafish embryos injected with morpholinos. One- or two-celled embryos were injected with 4 ng of S2-SP1 and Mcon MO (control). WT was injected with RNase-free water. Neuromast hair cells were measured with TUNEL and changes in apoptotic signals in the anterior and posterior lateral neuromasts occurred (scale bars = 10 μm). (B) Statistical analysis of the apoptotic hair cells in anterior and posterior lateral neuromasts in different types of embryos at 120hpf, including WT, S2-SP1 and Rescue, in order to exclude the possibility that the apoptosis hair cells of MO knockdown zebrafish were due to off-target MO toxicity, we co-injected S2-SP1 with p53-MO. ***P<0.001.

To confirm that the decreased number of hair cells is related to apoptosis, neuromast hair cells were detected with Tunel in 120hpf zebrafish larvae, nuclei were stained with DAPI. Both hair cells in the anterior lateral line and posterior lateral line neuromasts of morphants were detected have apoptotic signals (Fig 6A), and the average number of apoptotic hair cells per anterior neuromast was ≈10.7±1.3 in S2-SP1 zebrafish, and per posterior neuromast was ≈11.5±2.2 in S2-SP1 zebrafish, but apoptosis was nearly undetectable in WT zebrafish hair cells. To exclude the possibility that the otic disorder and decreased hair cells of MO knockdown zebrafish were due to off-target MO toxicity, we co-injected S2-SP1 with p53-MO, the results show no obvious difference between S2-SP1 single injected and S2-SP1coinjected with P53-MO. Rescue of S2-SP1-induced cell death was achieved by co-injection of *SLC26A2* mRNA.

### 3.6 Decreased numbers of hair cells in 120hpf larvae

**Fig 7:**
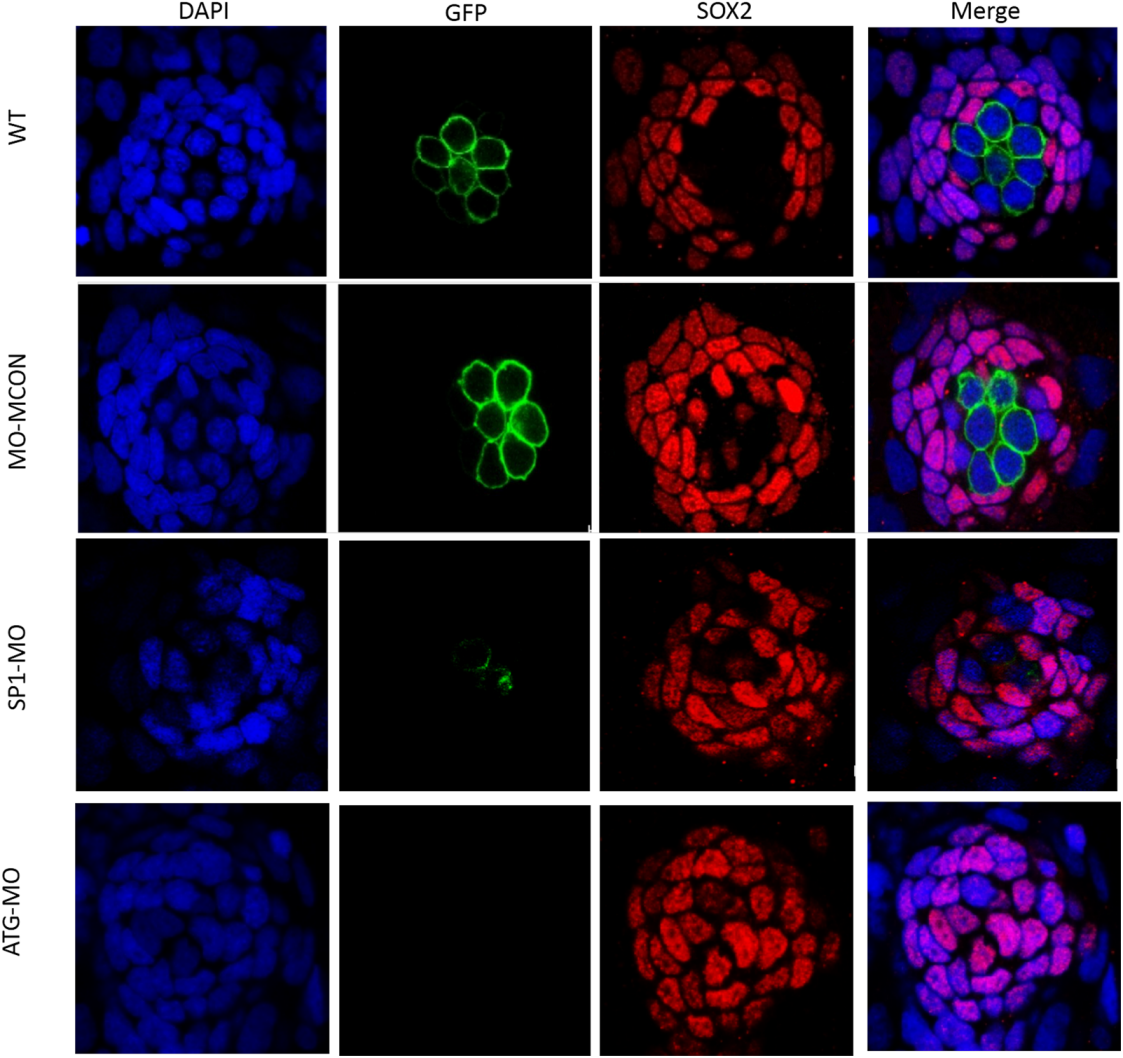
Numbers of supporting cells are unaffected in morphants, while numbers of hair cells decrease in morphants. Morphology of 120hpf Tg(Brn3c:mGFP) S356T transgenic zebrafish expressing GFP in hair cells under control of the POU4F3 promoter embryos injected with morpholinos. One- or two-celled embryos were injected with 4 ng of S2-SP1 and Mcon MO (control). WT was injected with RNase-free water.

To investigate whether the hair cells loss is accompanied by the loss of supporting cells, we injected S2-SP1 MO into Tg (Brn3c:GFP), and co-stained the 120hpf embryos with Sox2 antibody. Our results suggest that the S2-SP1 morphants have less hair cells without significantly affecting the numbers of supporting cells (Fig 7).

### 3.7. Hearing and swimming in *Slc26a2*-morphant zebrafish

The inner ear is important for zebrafish hearing and balance. Because of the predominant defects in inner ears of MOs knockdown zebrafish, swimming behaviour was investigated here. 120hpf WT larvae usually swam or rested with their backs facing upward and each fish tended to swim one depth. However, 120hpf MO knockdown zebrafish larvae usually remained stationary and rested in abnormal positions: they swam up and down or in circles. This abnormal swimming behaviour indicated a defective balance system.

**Fig 8:**
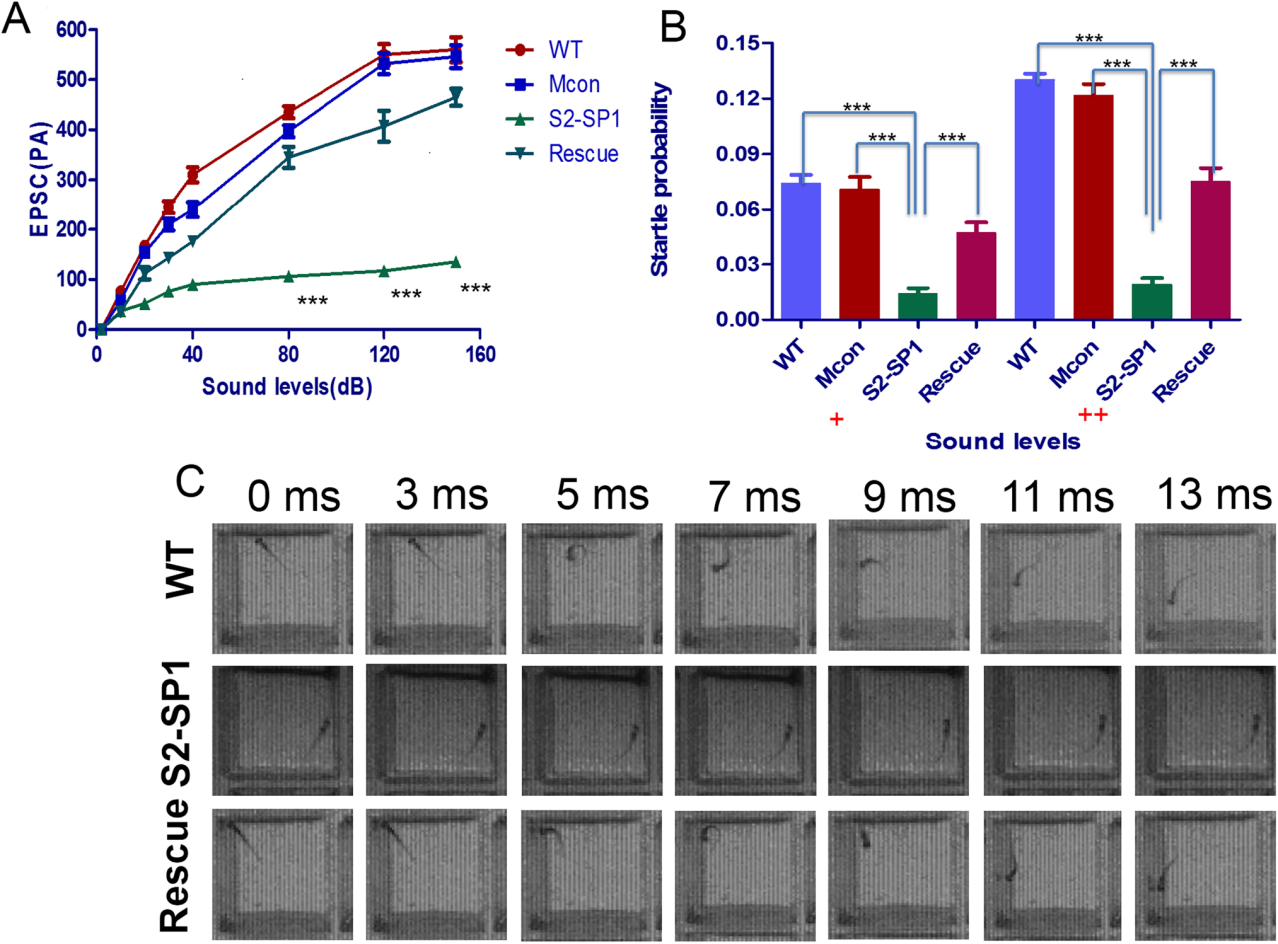
Hearing disability in S2-SP1 zebrafish at 120hpf. M-cells was recorded under sound stimulation (500 Hz, 10 ms). (A) Examples of the recorded EPSC in wild-type (WT), S2-SP1 konckdown, Mcon and rescued individuals. The sound stimuli (80 dB) lasted 10 ms, during which hair cell signals could be induced several times so that a single M-cell could receive input from many different hair cells through ganglion cells because multiple action potentials in the ganglion cells could be produced., As a result, multiple peaks were produced and the EPSC amplitudes were shown for different sound levels. (B) Average C-startle response probability. For each group, 50 larvae were tested. Sound intensities are designated with + and ++ because sound was applied using different units in the M-cell recording and startle response experiments. ***P<0.001. (C) Examples of C-shaped startle responses of different larvae types in response to 500 Hz frequency, 10 ms duration. WT and Mcon-MO larvae usually swam or rested with their backs facing upward, and they tended to swim at one depth. The typical escape response of WT larva was initiated 5–11 ms after sound stimulation, However, S2-SP1 larvae usually remained stationary and rested in abnormal positions. They swam up and down or in circles. Abnormal swimming behaviour of S2-SP1 knockdown zebrafish indicated defective balance. Some S2-SP1 larvae had no response to outside sound stimulation. After rescue, S2-SP1 zebrafish regained their hearing.

To assess hearing loss of 120hpf morphants, the electrophysiological experiments to record the excitatory postsynaptic currents (EPSC) in the Mauthner cells (M-cells) of 120hpf larvae was performed and the fast escape reflex, a C-shaped startle response mediated predominately by M cells was measured using near-field pure tone stimulation with two different sound intensities. Both wild-type and S2-SP1 konckdown siblings showed robust EPSC in the M-cells after the sound stimulation (Fig 8A). Compared with wild-type siblings, the S2-SP1 larvae exhibited significant decrease in the amplitudes when the sound intensity was ≥80 dB (P <0.01) and the larvae rescued with human *SLC26A2* mRNA had no statistically significance compared with WT zebrafish. These data indicate that the hearing of the S2-SP1 knockdown was severely impaired. The results showed that the C-startle response of S2-SP1 knockdown was statistically different from their WT siblings, the probability of the C-startle response between WT and S2-SP1 larvae was distinguishable for either sound intensity, especially for zebrafish with small otoliths. With human homologous mRNA rescue, about half of the morphants regained some hearing ability, audio stimulus sensitivity was greatly increased, and swimming postures normalized (Fig 8).

## Discussion

In 2013, the World Health Organization estimated that 360 million people worldwide live with disabling hearing loss and as the population ages, the global burden of disease attributable to deafness then increases [32]. Traditional methods of screening new disease-causing genes are expensive and time-consuming, but biotechnology offers unique methods for finding new genes.

Here 77 reported nonsyndromic deafness genes were classified using their biological characteristics, and 2,455 GO terms were added through the analysis and simulation of the Funckenstein algorithm. Then performance was evaluated using cross-validation and examination of literature associated with top-scoring novel predictions. Data indicate that more than 300 candidate genes were predicted using the features of 16 gene types and 6 types of relationships among different genes. Then candidate genes were ranked according to a quality rating system. Results showed that top-ranked predicted genes were related to the auditory system. After application of selection criteria, *SLC26A2* was chosen for further study.

*SLC26A2*, a diastrophic dysplasia sulphate transporter gene, encodes a transmembrane protein that transports sulphate into chondrocytes to maintain adequate proteoglycan sulphation [12, 33]. As bicarbonate can be transported across cell plasma membranes by anion exchangers of the SLC26 gene families, and the main constituent part of otoliths is dense calcium carbonate crystals. Semicircular canal is full of endolymph which has many ions, and it is speculated that deficiency of *slc26a2* contributes to the dysfunction of otoliths and semicircular canal. Mutations in this gene are responsible for four recessively inherited chondrodysplasias, including diastrophic dysplasia, multiple epiphyseal dysplasia, atelosteogenesis type 2, and achondrogenesis 1B

[34]. More than 47 mutations have been observed in this gene, but until now, in all the many clinical case reports of *SLC26A2*-related chondrodyspasia, no deafness or hearing loss has been observed to cosegregate with disease. Whole-mount *in situ* hybridization (WISH) data indicated that *slc26a2* is highly expressed in the otic vesicle and in the primordium of the posterior lateral line of zebrafish, suggesting that *slc26a2* might be key to hearing development. To confirm that disruption of normal *SLC26A2* function can cause hearing loss. *slc26a2* was knocked down using antisense oligonucleotides. *slc26a2*-knockdown fish displayed abnormal phenotypes, including abnormal otoliths, smaller inner ear, and malformed semicircular canals in the inner ear, and posterior lateral neuromasts deposition was significantly affected in *slc26a2*-knockdown fish. On average, there were far fewer L1-L8 lateral neuromasts than in WT fish, indicating that there were fewer functional hair cells per neuromast.

*Slc26a2* was specifically expressed in the lateral line primordium. During development of the lateral line system, the primordium arises from the lateral line placode [35]. Then the primordium migrates caudally. This is followed by the formation of proneuromasts. Neuromasts originate from the placode and move along the horizontal myoseptum to the posterior end of the body [36]. It is here speculated that *slc26a2* may be vital to the migration process, so knockdown of *slc26a2* in zebrafish may affect placode growth and lateral line primordium migration and neuromasts deposition, and this may reduce the number of neuromasts.

In most humans with profound hearing loss, irreversible hair cell loss due to apoptosis leaves them unable to generate electrical activity in the auditory system [37]. To elucidate the mechanism behind the decrease in the number of hair cells in the lateral neuromast, TUNEL was used to measure hair cell viability. Data show that hair cells of lateral neuromasts in *slc26a2* knockdown zebrafish had stronger apoptotic signals. During our study, we found that the hair cells death after injecting *slc26a2* MO is tissue-specific and not off target based on control MO results. However, we can’t rule out the possibility that the apoptosis of hair cells still relies on the p53 pathway.

Many genes encoding different transporters and channels are highly expressed in the ear and participate in maintaining unique fluid homeostasis [38]. Endolymphatic pH homeostasis involves H^(+)-^ATPase and Cl^-^/HCO_3_^-^ exchangers including pendrin [39]. Maintenance of appropriate fluid homeostasis is important, as evidenced by the fact that mutations in genes such as the solute carrier transporter gene *SLC26A4* (pendrin) lead to prelingual deafness. Pendrin appears responsible for mediating Cl^-^/HCO_3_^-^ exchange in the inner ear. It is also involved in endolymphatic fluid conditioning, presumably because of HCO_3_^-^ secretion. In this way, it modifies inner ear acid-base homeostasis [40]. *Slc26a2* is an SO4^2-^/Cl^-^/OH^-^ exchanger [12]. It is here speculated that *slc26a2* contributes to the ionic endolymph environment and creates an osmotic gradient to establish the ion potential in developing hair cells. In this way, defective *slc26a2* destroys this osmotic gradient and ion potentials and may change the acid-base homeostasis of the inner ear, destroying hair cell depolarization, leading to hair cell apoptosis.

To confirm the existence of hearing impairment in zebrafish, the C-shaped startle response, which is mediated predominately by M cells, was measured using near-field pure tone stimulation at two sound intensities. Consistent with MO knockdown results, ≈80% of 7-day-old morphants did not respond to acoustic stimuli or were unable to remain upright while swimming. The C-startle response of S2-SP1 knockdown fish was statistically different from WT siblings. These data support the idea that down-regulation of *slc26a2* could influence the hearing systems of zebrafish and cause hearing loss.

Neural apoptosis has been reported to be induced by MO and the similarity to the phenotype induced by apparent p53 up-regulation suggested that off-target effects of MO could induce the p53 apoptotic pathway [23–24]. To exclude abnormal features of inner ears in S2-SP1 knockdown zebrafish caused by off-target effects, S2-SP1 and p53-MO were co-injected into zebrafish. However, p53 MO was not found to attenuate the otic abnormal phenotype induced by MO, as shown by acridine orange staining. This confirmed that the abnormal phenotype caused by MO is a specific target of *slc26a2*.

Because the human *SLC26A2* sequence is closely homologous with zebrafish according to multiple sequence alignment and phylogeny evolutionary analysis, the specificity of the S2-SP1-induced abnormal phenotype was measured in the inner ear using overexpression of a human *SLC26A2* RNA construct. It was here observed that S2-SP1 knockdown phenotypes can be corrected and that the abnormal phenotype in the inner ear is not induced by off-target effects but rather by specific effects of MO. The abnormal phenotype was partially rescued using highly homologous human mRNA, which suggests that the *SLC26A2* gene in zebrafish is closely related to the hearing loss gene in humans.

Here, a combination of morpholino oligonucleotide knockdown technology and bioinformatics techniques was shown to be a feasible way of discovering and acquiring novel genes associated with deafness.

More work is needed to determine whether *SLC26A2* is a deafness gene. In particular large clinical deafness pedigrees and sporadic cases must be exploited to identify differences in deaf patients’ DNA by sequencing whole exons of *SLC26A2*.

## Supporting Information

S1 Fig AO straining in wildtype zebrafish, S2-SP1 morphant, S2-SP1 co-injected with p53-morpholino zebrafish. AO straining is shown in S1 Fig. No obvious apoptotic cells were observed among WT, Mcon, and S2-SP1 knock-down zebrafish. There were no differences between S2-SP1 and p53 treated S2-SP1.
(TIF)

S2 Fig *SLC26A2* is highly conserved among different vertebrate species. The multiple sequence alignment and phylogeny evolutionary analysis of slc26 family members, which showed that *SLC26A2* is highly evolutionarily conserved among different vertebrate species.
(TIF)
