# Supplementary figures and images for: Solute Carrier Family 26 Member a2 (slc26a2) Regulates Otic Development and Hair Cell Survival in Zebrafish
Source: PLoS One. 2015 Sep 16;10(9):e0136832. doi: 10.1371/journal.pone.0136832 (PMC4573323; doi:10.1371/journal.pone.0136832)

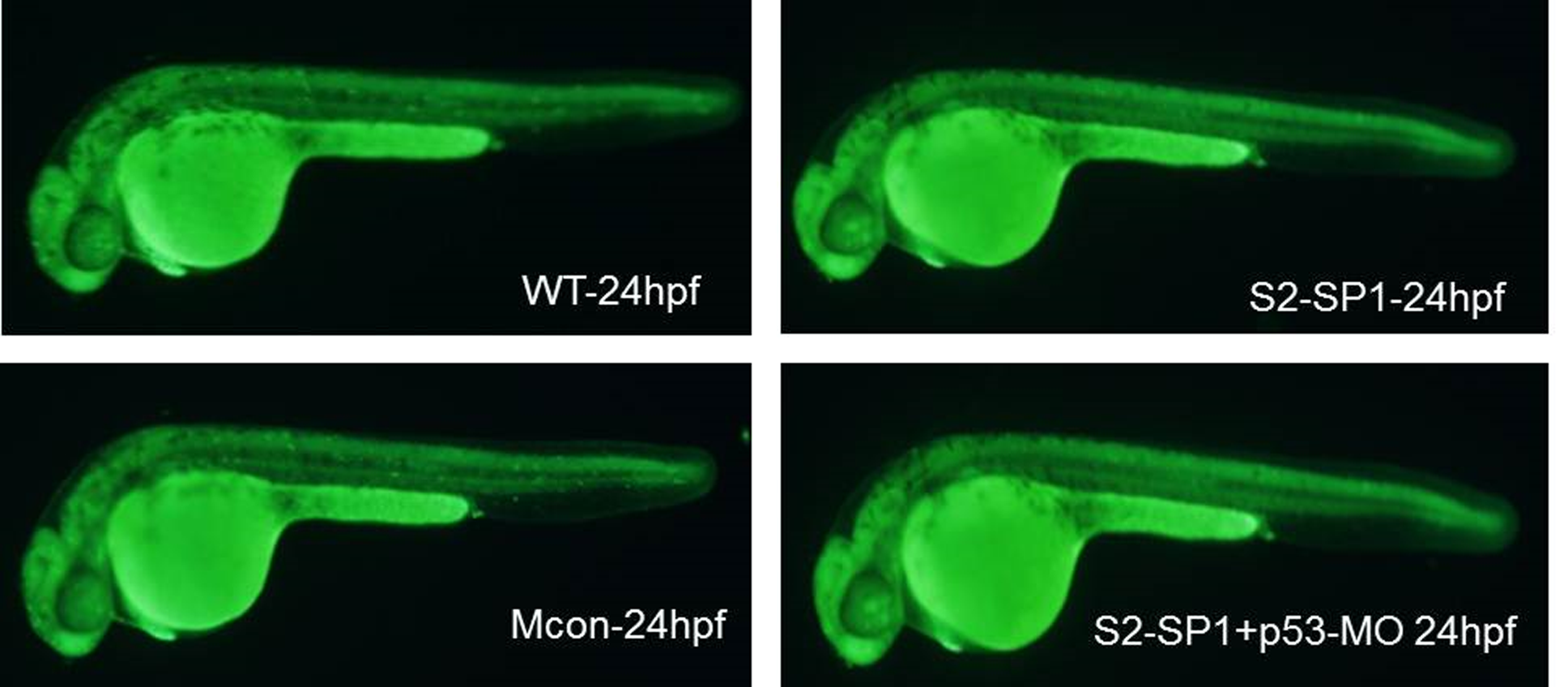

Supplement: S1 Fig — AO straining is shown in S1 Fig. No obvious apoptotic cells were observed among WT, Mcon, and S2-SP1 knock-down zebrafish. There were no differences between S2-SP1 and p53 treated S2-SP1. (TIF) [file pone.0136832.s001.tif]

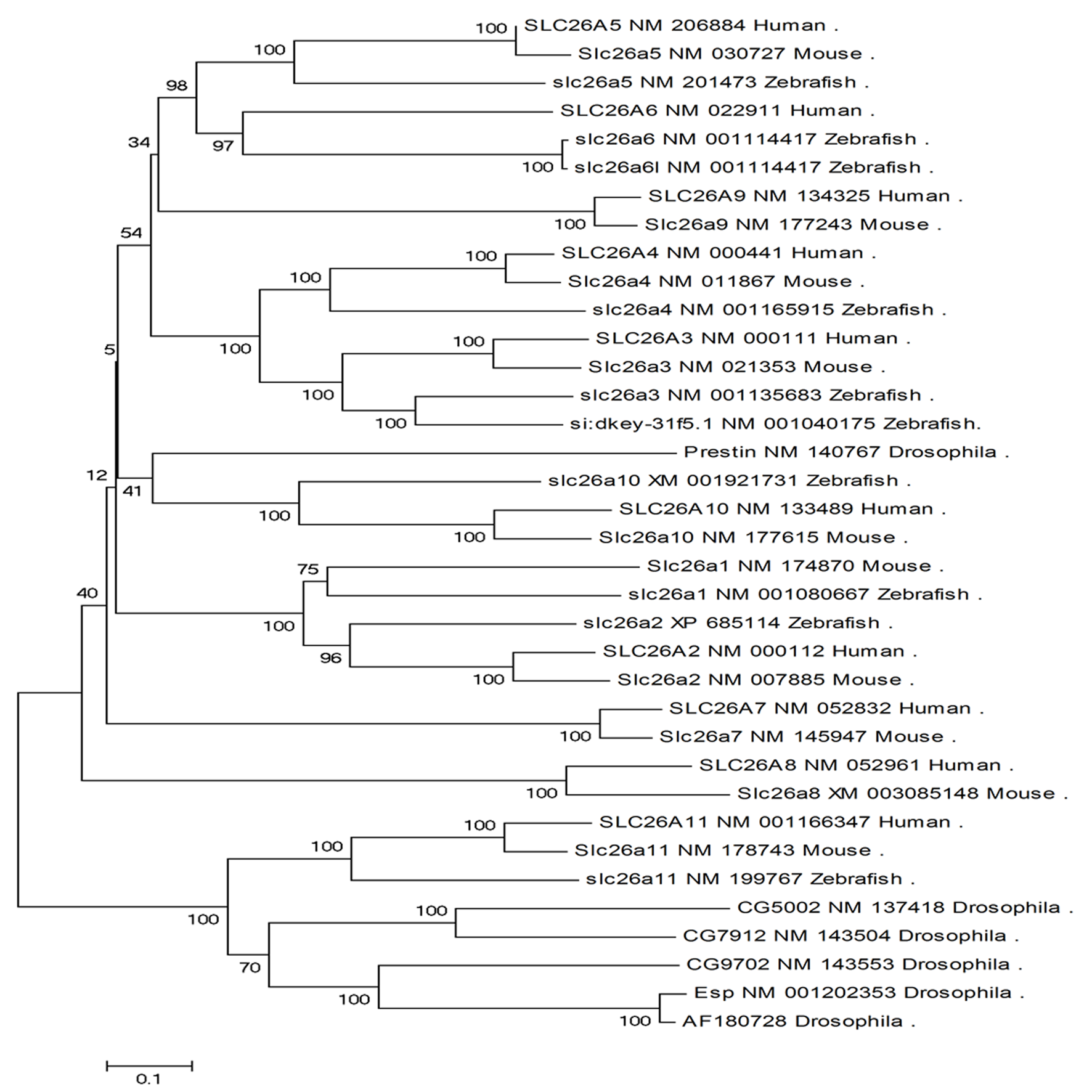

Supplement: S2 Fig — The multiple sequence alignment and phylogeny evolutionary analysis of slc26 family members, which showed that SLC26A2 is highly evolutionarily conserved among different vertebrate species. (TIF) [file pone.0136832.s002.tif]
